# Who Wants to Be Alone? Antecedents of Motivation for Solitude in Adulthood

**DOI:** 10.1093/geroni/igaa057.1535

**Published:** 2020-12-16

**Authors:** Jing Yuan, Daniel Grühn

**Affiliations:** North Carolina State University, Raleigh, North Carolina, United States

## Abstract

Objectives: As an inevitable part of daily life, solitude has both positive and negative consequences which are moderated by one’s motivation for solitude. Self-determined motivation correlates with few psychological risks, whereas other-determined motivation correlates with higher risks (e.g., loneliness, depression, lower well-being). However, little is known about the antecedents of different motivations for solitude. The purpose of this study is to investigate the antecedents of motivation for solitude in a sample with younger, middle-aged, and older adults. Methods: We recruited 468 participants from Amazon Mechanical Turk and Introduction to Psychology class (age range: 17-70, M = 30.7, 50.4 % females). Preference and motivation for solitude were measured with the Preference for Solitude Scale and Motivation for Solitude Scale-Short Form. Age, sex, marital status, education level, living arrangement, instrumental activities of daily living (IADLs), civic engagement, social contact frequency, introversion, and empathy were measured as antecedents. Results: People with older age, higher empathy for fictional characters, lower personal distress, higher introversion, and females tended to have higher general preference for solitude. People with higher empathy for fictional characters tended to have higher self-determined motivation. People with higher empathy for fictional characters, lower empathic concern, higher personal distress, higher IADLs, and higher introversion were more likely to have higher other-determined motivation. Discussion: A person with an introverted personality, functional limitation, more negative empathic reactions towards others are likely to have maladaptive motivation for solitude and may need intervention. Future research should further investigate other antecedents for self-determined motivation.

